# 

**Published:** 1967-12

**Authors:** 


					m Svl
December 1907
INCORPORATING THE MEDICAL JOURNAL OF THE SOUTH WEST
82 (iv) No 306
A

				

